# Clinicopathological and Prognostic Value of USP22 Expression in Gastric Cancer: A Systematic Review and Meta-Analysis and Database Validation

**DOI:** 10.3389/fsurg.2022.920595

**Published:** 2022-06-16

**Authors:** Yuhang Wang, Zirui Jia, Jiacheng Gao, Tingting Zhou, Xiangwen Zhang, Guo Zu

**Affiliations:** ^1^ Department of General Surgery, The Dalian Municipal Central Hospital Affiliated of Dalian Medical University, Dalian, China; ^2^ Department of Graduate School, Dalian Medical University, Dalian, China; ^3^ Department of Neurology, The First Affiliated Hospital of Dalian Medical University, Dalian, China

**Keywords:** USP22, gastric cancer, overall survival, clinicopathological parameters, meta-analysis

## Abstract

**Background:**

It has been reported that there is a correlation between the level of ubiquitin-specific protease 22 (USP22) and the clinicopathological parameters and prognosis of gastric cancer (GC) patients, but the conclusions are inconsistent. Hence, a meta-analysis must be conducted to clarify the relationship between USP22 expression and clinicopathological and prognostic value of GC patients to provide more accurate evidence.

**Methods:**

According to the predetermined selection criteria, systematic file retrieval was performed. The hazard ratio (HR) or odds ratio (OR) and its 95% confidence interval (CI) were used to evaluate the relationship between USP22 expression and clinicopathological and prognostic value of GC patients.

**Results:**

In a total of 802 patients, those with GC were finally included in 6 studies. The pooled results demonstrated that the expression of USP22 was significantly increased in GC tissues compared with control tissues (OR = 9.947, 95% CI, 6.074–16.291, *P* = 0.000), and USP22 expression was related to lymph node metastasis (OR = 2.415, 95% CI, 1.082, *P* = 0.031), distant metastasis (OR = 3.956, 95% CI, 1.365–11.464, *P* = 0.011) and TNM stage (OR = 2.973, 95% CI, 1.153–7.666, *P* = 0.024). Nevertheless, the expression of USP22 was not correlated with gender (OR = 1.202, 95% CI, 0.877–1.648, *P* = 0.253), age (OR = 1.090, 95% CI, 0.811–1.466, *P* = 0.568), tumor size (OR = 0.693,95% CI, 0.348–1.380, *P* = 0.297), tumor differentiation (OR = 1.830, 95%CI, 0.948–3.531, *P* = 0.072) and depth of invasion (OR = 2.320, 95% CI, 0.684–7.871, *P* = 0.177). Moreover, a high expression of USP22 predicted a poor overall survival (OS) in GC patients (HR = 2.012, 95% CI, 1.522–2.658, *P* = 0.000). The database of Kaplan–Meier plotter confirmed that a high expression of USP22 was correlated with poor prognostics in GC patients (HR = 1.41, 95% CI, 1.18–1.68, *P* < 0.01).

**Conclusion:**

USP22 overexpression in GC tissues is positively related to lymph node metastasis, distant metastasis and TNM stage and indicates a poor clinical outcome of GC patients, but it is not associated with age, gender, depth of invasion, tumor differentiation and tumor size of GC patients.

**Systematic Review Registration**: https://www.crd.york.ac.uk/prospero/, identifier: 338361.

## Introduction

Gastric cancer (GC), as a common malignant tumor, is the third leading cause of cancer death and ranks fifth in terms of cancer incidence (1). Although the treatment of GC has seen progress in the form of surgical technology, radiotherapy and chemotherapy, GC remains an important health issue worldwide. Most patients will already be in an advanced stage when they are diagnosed with GC, and the prognosis of advanced GC is extremely poor (2). Therefore, it is of great significance to find effective biomarkers to accurately determine the clinicopathological significance and prognosis of GC patients.

Ubiquitin-specific peptidase 22 (USP22), one of the highly conserved ubiquitin hydrolases, is involved in the formation of transcriptional protein acetylation composites. USP22 regulates gene transcription by catalyzing the removal of mono-ubiquitination of histones H2A and H2B (3). In recent years, USP22 has been reported as a member of 11 “Death-from-Cancer” genes (4, 5). It has been shown that USP22 is overexpressed in many solid tumors, for example, bladder cancer, breast cancer and colorectal cancer, which means that it is a potential cancer biomarker (6–8). Recently, an increasing number of evidence has also demonstrated that USP22 is overexpressed in GC (9–14). However, researchers have different conclusions about whether USP22 expression is related to clinicopathological parameters and clinical outcomes in patients with GC. Yang et al. indicated that a high expression of USP22 is correlated with prognosis and tumor differentiation of GC patients but not with tumor size (9). However, Liu et al. reported that in GC patients, USP22 expression is positively related to tumor size but not to tumor differentiation and prognosis (10). In order to resolve the current controversies, we conducted a systematic study and performed a meta-analysis on the correlation between USP22 and clinicopathological features and prognosis of GC patients.

## Methods

### Search Studies

In order to collect all relevant data, we made a comprehensive survey on the Web of Science, PubMed, Embase, the Cochrane Library, CNKI and WanFang database, from inception to January 1, 2022. The following terms were used as keywords: (“gastric cancer” or “stomach cancer” or “gastric carcinoma”) and (“USP22” or “Ubiquitin-specific peptidase 22”). Furthermore, the identified studies were manually inspected to improve the integrity of the eligible papers.

### Inclusion and Exclusion Criteria

Articles must be in accordance with the following standards to be adopted: (1) the patients were definitively diagnosed with GC by pathology; (2) the relationship between USP22 expression and the clinicopathological parameters (age, gender, TNM stage, tumor differentiation, etc*.*) of GC patients was investigated; (3) the articles described the association between USP22 expression and medical outcomes in GC patients, including overall survival (OS); and (4) sufficient information was used to estimate the 95% CIs.

If the following criteria are met, the studies are excluded: (1) cell experiment; (2) case-only studies; (3) reviews, meta-analysis, letters and case reports; (4) non-original research; (5) other cancers; (6) repeat research based on the same database or patients; and (7) the patients received radiotherapy and chemotherapy before operation.

### Data Extraction

The following information was identified and screened by two authors (Wang and Jia) from each eligible publication according to the prescribed standards: first author, publication year, country, study period, essay method, age, gender, tumor invasion, lymph node metastasis, distant metastasis, TNM stage, tumor differentiation, tumor size and OS. During this period, the different opinions were resolved by discussing all the contents with the third author (Gao) and reaching a consensus. The Newcastle–Ottawa Scale (NOS), with a score range of 0–9, was used to assess and score the quality of the study. When the score was 6 or higher, it was considered to be a high-quality literature.

### Database Validation and Bioinformatics Analysis

The Kaplan–Meier Plotter database (http://www.kmplot.com) was used to analyze the effect of USP22 on OS in GC patients. The data of GC patients with USP22 expression was extracted from TCGA, GEO and the EGA database (https://www.cancer.gov/tcga).

### Statistical Analysis

We extracted raw data from eligible studies to obtain combined odds ratio (OR), hazard ratio (HR) and 95% confidence interval (CI), which were used to evaluate the effect of USP22 on clinicopathological parameters and OS in patients with GC. Statistical heterogeneity among studies was analyzed by using the Q-test and *I*^2^ test. If the p-value was less than 0.05 or the *I*^2^ value was greater than 50%, then significant heterogeneity existed in the data and the random effects model would be adopted. Otherwise, the fixed effects model would be used. In this meta-analysis, Stata 16.0 was used to analyze the extracted data.

## Results

### Characteristics of Studies Included in Meta-Analysis

**Figure 1:**
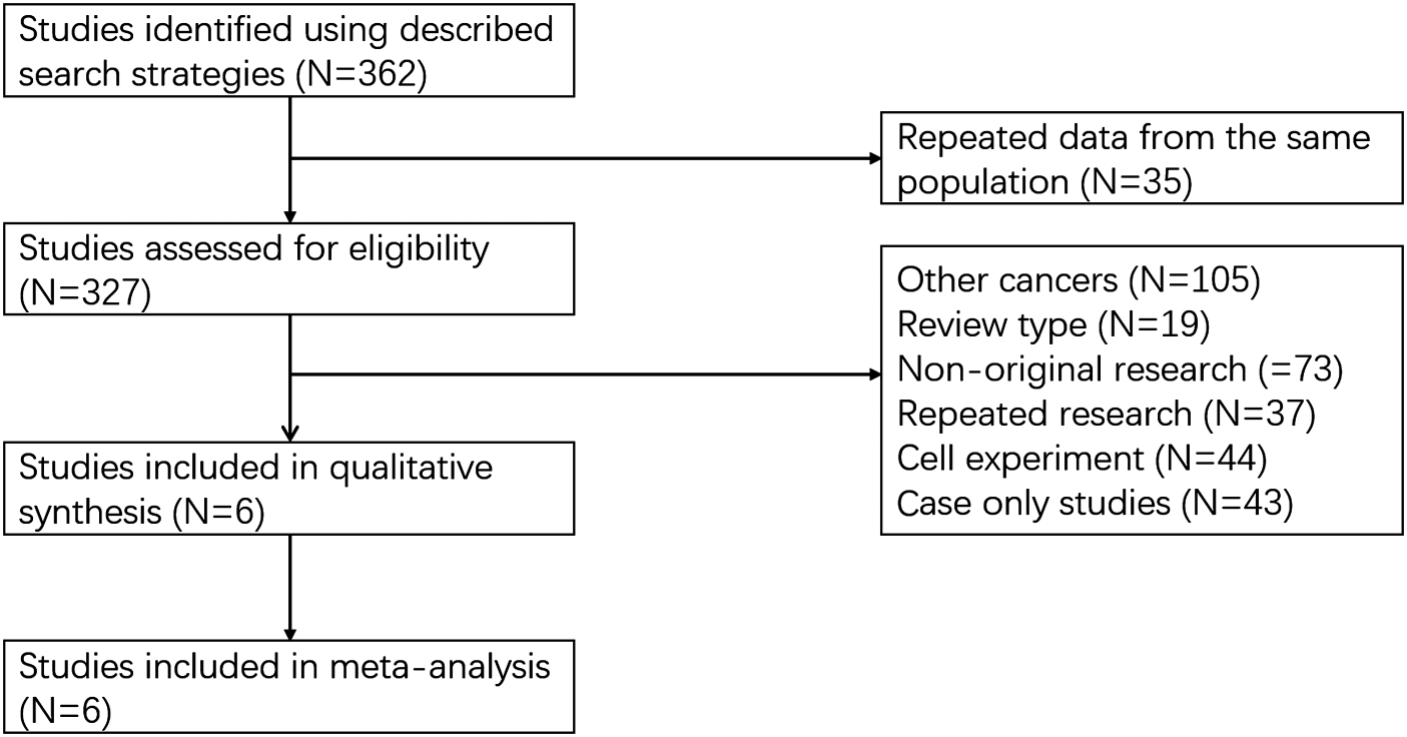
Flow chart of research data screening.

A total of 362 articles were selected in the relevant database. The recorded data of 35 articles were repeated from the same population. Among the 327 articles, 321 articles were excluded, and these contained other cancers (*N* = 105), review type (*N* = 19), non-original research (*N* = 73), repeat research (*N* = 37) and cell experiment (*N* = 87). Six articles were proved to be within the scope of the study and the average NOS score was 7 (Figure 1) (9–14). In total, 802 patients with GC were enrolled in our study. The basic information on these datasets is summarized in Table 1.

**Table 1:** Main characteristics and results of each study.

No.	Author	Year	Journal	Country	Study period	Sample size	M/F	Method	NOS
1	Liu	2019	Aging	China	2004.1–2008.12	186	119/67	IHC	8
2	Lim	2020	Cancer Cell Intemational	China	2007.5–2008.2	88	65/23	IHC	7
3	Zheng	2019	Patholoay-Research and Practic	China	Not report	84	50/34	IHC	6
4	Deng	2011	Journal of Abdominal Surgery	China	2008.1–2009.7	100	66/34	IHC	7
5	Yang	2011	Cell Biochem Biophys	China	2004.1–2005.11	219	162/57	IHC	6
6	Yu	2016	Journal of Harbin Medical University	China	2004.1–2004.12	125	82/43	IHC	8

### The Expression of USP22 in GC and Control Tissues

**Figure 2:**
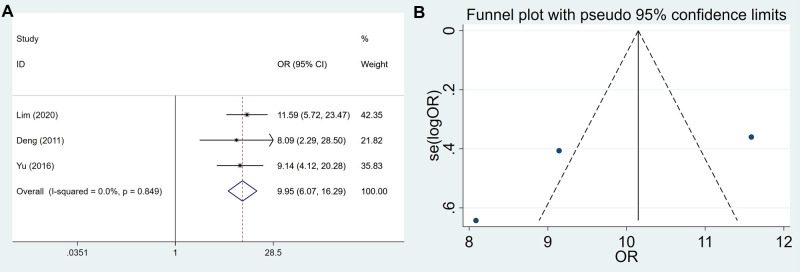
Forest plots and funnel plots for the USP22 expression between GC and control tissues. (**A**) forest plots and (**B**) funnel plots.

A total of 313 GC tissues and 158 control tissues were included to assess USP22 expression in GC patients. The results showed that the expression of USP22 was significantly increased in GC tissues compared with control tissues (OR = 9.947, 95% CI, 6.074–16.291, *P* = 0.000) (Figure 2A). There was no significant bias in the expression of USP22 of GC tissues and control tissues (Egger’s test, *P* = 0.413) (Figure 2B).

### USP22 Expression and Clinicopathological Factors

**Figure 3:**
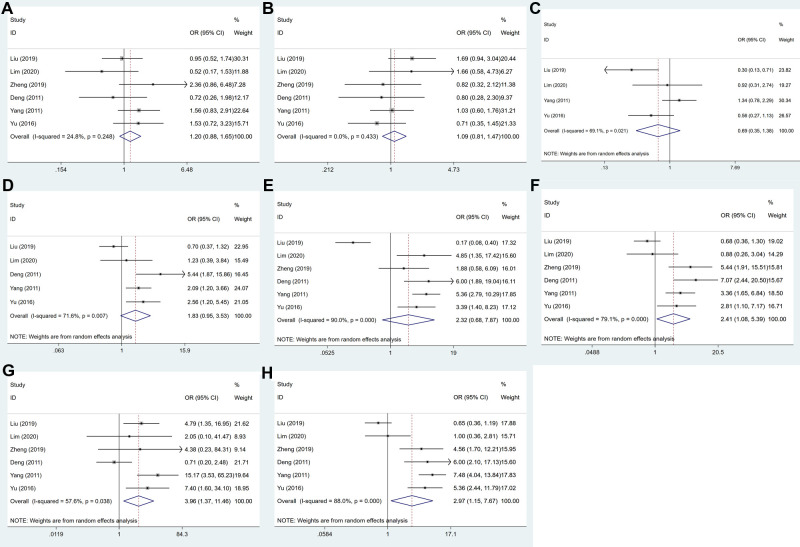
Forest plots for the association of USP22 expression with clinicopathological parameters: (**A**) gender, (**B**) age, (**C**) tumor size, (**D**) tumor differentiation, (**E**) depth of invasion, (**F**) lymph node metastasis, (**G**) distant metastasis and (**H**) TNM stage.

The association between USP22 expression and clinicopathological parameters is shown in Figure 3 and Table 2. After systematic analysis, it was found that USP22 expression was not correlated with gender (OR = 1.202, 95% CI, 0.877–1.648, *P* = 0.253) (Figure 3A), age (OR = 1.090, 95% CI, 0.811–1.466, *P* = 0.568) (Figure 3B), tumor size (OR = 0.693,95% CI, 0.348–1.380, *P* = 0.297) (Figure 3C), tumor differentiation (OR = 1.830, 95%CI, 0.948–3.531, *P* = 0.072) (Figure 3D) and depth of invasion (OR = 2.320, 95% CI, 0.684–7.871, *P* = 0.177) (Figure 3E). However, the expression of USP22 was correlated with lymph node metastasis (OR = 2.415, 95% CI, 1.082, *P* = 0.031) (Figure 3F), distant metastasis (OR = 3.956, 95% CI, 1.365–11.464, *P* = 0.011) (Figure 3G) and TNM stage (OR = 2.973, 95% CI, 1.153–7.666, *P* = 0.024) (Figure 3H).

**Table 2:** The association between USP22 expression and clinicopathological factors in GC.

Factor	Number of studies	Number of patients	Pooled OR (95% CI)	*P* value	Heterogeneity		
					*I*^2^ (%)	*P* value	Model
Gender (male vs. female)	1,2,3,4,5,6	802	1.202 (0.877–1.648)	0.253	24.8	0.248	Fixed
Age (≥60 vs. <60)	1,2,3,4,5,6	802	1.090 (0.811–1.466)	0.568	0.00	0.433	Fixed
Tumor size (≥5 vs. <5 cm)	1,2,5,6	618	0.693 (0.348–1.380)	0.297	69.1	0.021	Random
Tumor differentiation (well vs. poor)	1,2,4,5,6	718	1.830 (0.948–3.531)	0.072	71.6	0.007	Random
Depth of invasion (T_3-4_ vs. T_1-2_)	1,2,3,4,5,6	802	2.320 (0.684–7.871)	0.177	90.0	0.000	Random
Lymph node (yes vs. no)	1,2,3,4,5,6	802	2.415 (1.082–5.389)	0.031	79.1	0.000	Random
Distant metastasis (yes vs. no)	1,2,3,4,5,6	802	3.956 (1.365–11.464)	0.011	57.6	0.038	Random
TNM stage (III-IV vs. I-II)	1,2,3,4,5,6	802	2.973 (1.153–7.666)	0.024	88.0	0.000	Random

### Publication Bias of USP22 Expression and Clinicopathological Factors

**Figure 4:**
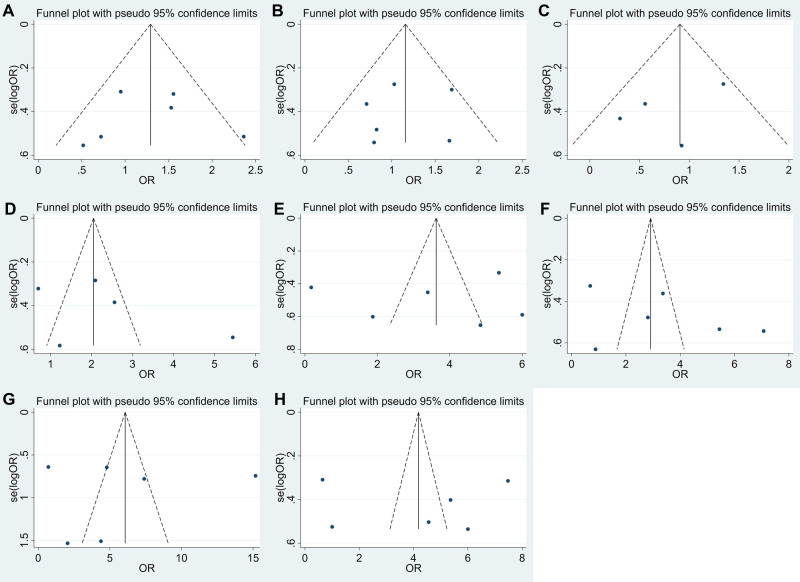
Funnel plots for the association of USP22 expression with clinicopathological parameters: (**A**) gender, (**B**) age, (**C**) tumor size, (**D**) tumor differentiation, (**E**) depth of invasion, (**F**) lymph node metastasis, (**G**) distant metastasis and (**H**) TNM stage.

Egger’s test was selected for evaluating the publication bias. The conclusions drawn from the funnel plots suggested that there was no obvious bias in gender (*P* = 0.635) (Figure 4A), age (*P* = 0.660) (Figure 4B), tumor size (*P* = 0.427) (Figure 4C), differentiation (*P* = 0.599) (Figure 4D), invasion (*P* = 0.937) (Figure 4E), lymph node metastasis (*P* = 0.358) (Figure 4F), distant metastasis (*P* = 0.851) (Figure 4G) and TNM stage (*P* = 0.740) (Figure 4H).

### USP22 Expression and OS of GC Patients

**Figure 5:**
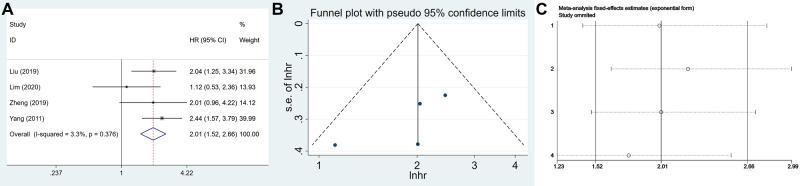
The association of USP22 expression with the OS of GC patients: (**A**) forest plot, (**B**) funnel plots and (**C**) sensitivity analysis.

A total of 702 patients were included from four eligible articles, which provided us with data to evaluate the relationship between USP22 and OS. The data from the forest plot indicated that a high USP22 level predicted poor outcome in GC patients (HR = 2.012, 95% CI, 1.522–2.658, *P* = 0.000) (Figure 5A). No significant bias was found in the prognosis of patients with GC (Egger’s test, *P *= 0.227) (Figure 5B).

Sensitivity analysis was performed to assess the stability of the meta-analysis results. After sequentially deleting each study, the results suggested no change in the overall results of OS, which meant that the result of our meta-analysis was highly stable (Figure 5C).

### Database Validation and Bioinformatics Analysis

**Figure 6:**
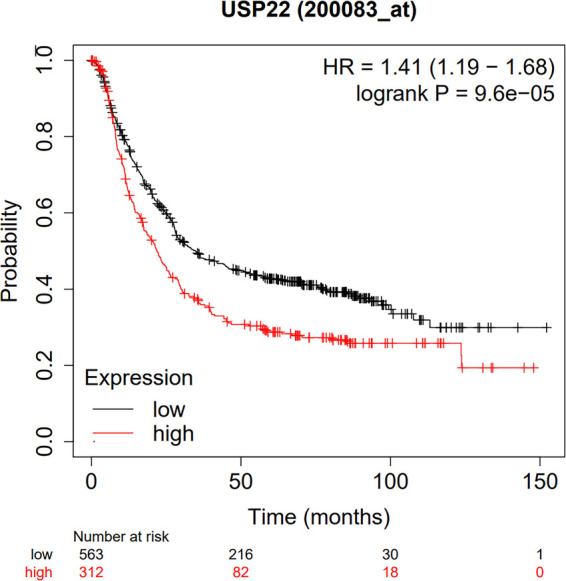
Database validation to explore the relationship between USP22 expression and the OS of GC patients based on TCGA, GEO and EGA.

According to the database of the Kaplan–Meier plotter, the results suggested that USP22 overexpression expression was significantly related to poor OS (HR = 1.41, 95% CI, 1.18–1.68, *P* < 0 .01) (Figure 6).

We conducted a subgroup analysis on the relationship between USP22 and prognosis in patients with GC. The results showed that there was no difference in the diagnostic value of USP22 in different T, N and TNM stages of GC patients (Supplementary Figure S1).

## Discussion

GC, as a gastrointestinal malignancy, is one of the most common causes associated with cancer deaths worldwide (15). In the past century, the morbidity and mortality rates of GC in most countries dropped steadily. But due to the changes in the age of population and diet structure, the increase in work pressure and Helicobacter pylori infection, the incidence rate of GC is still high (16). Recently, suitable biomarkers for early detection and diagnosis of GC have drawn the attention of researchers. For example, USP22 has been reported to be associated with clinicopathological features and clinical outcomes in numerous studies, but it has proved controversial. In this meta-analysis, we reported that USP22 expression was closely related to lymph node metastasis, distant metastasis and TNM stage in GC patients but not to age, gender, depth of invasion, tumor differentiation and tumor size. The high expression of USP22 suggested poor OS in GC patients, which might be an indicator of poor prognosis of GC patients.

USP22 is a catalytic subunit that regulates gene transcription by removing monoubiquitination of histones H2A and H2B. In addition, USP22 is involved in regulating the function of multiple non-histone targets, which are correlated with cancer progression and poor prognosis (17). Recently, some researchers have reported that USP22 plays a vital role in regulating the cell cycle and driving transcription (18–20). It has been proved that USP22 is one of the significant biomarkers of cancer stem cells (21, 22). USP22 overexpression has been reported in several human cancers such as bladder cancer, colorectal cancer and oral squamous cell carcinoma and is related to the clinicopathological parameters and prognosis of many types of cancers (23–29). Moreover, several studies have reported that a high level of USP22 is thought to play a significant role in patients with GC (9, 10, 21–24). Researchers have used the transwell migration and invasion assays to analyze the effect of USP22 on cell motility, and the results revealed that the migration of USP22 silenced cells is significantly reduced (10). Other researchers have also detected the expression of USP22 in GC samples, and the results showed that USP22 protein expression levels are obviously upregulated in GC patients with lymph node metastasis. However, the correlation between USP22 expression and lymph node metastasis of GC patients is still contradictory (9). Lim et al. examined USP22 protein expression in 88 GC tissue samples to investigate the role of USP22 in GC and found no clear relationship between USP22 overexpression and lymph node metastasis of GC (21). Meanwhile, USP22 is reported to promote GC distant metastasis (9, 10, 23). However, Deng et al. showed that USP22 expression in GC tissues was not associated with distant metastasis. In addition, Lim et al. showed that the upregulation of the USP22 gene was not associated with the advanced TNM stage of GC, and these inconsistencies with other included studies might be due to the relatively small sample size of this study (9, 10, 22, 23, 24). In our meta-analysis, our results suggested that USP22 expression was correlated with lymph node metastasis, distant metastasis and the tumor TNM stage of GC patients.

Researchers have established tumor xenograft models in mice to evaluate the role of USP22 in tumor growth *in vivo*. They found that tumor growth was obviously reduced after the USP22 gene was knocked out (10). Furthermore, Liu et al. found that in GC patients, a high expression of USP22 was positively correlated with a tumor size of more than 5 cm (10). However, other authors indicated that no evidence of USP22 upregulation was found in GC patients (9, 11, 13). The authors reported that the expression level of USP22 was positively correlated with the T stage of GC (9–14). But Zheng et al. showed that the expression of USP22 was not related to the T stage of GC (12). The expression level of USP22 protein in GC tissue samples was negatively correlated with the degree of tumor differentiation (9, 10, 11, 13, 14). However, Lim et al. found no statistical difference after analyzing the relationship between USP22 expression and histological grade (11). Through a systematic study, our results suggested that USP22 expression was not associated with the depth of invasion, tumor differentiation and tumor size. However, the roles of depth of invasion, GC differentiation and tumor size for USP22 expression in GC patients need to be analyzed in a larger and randomized controlled trial sample. Therefore, further studies should be performed to detect the signal transduction pathway and the corresponding regulation mechanism of USP22 in GC cells and to further understand the role of USP22 in tumor genesis and the development of GC.

USP22 belongs to the ubiquitin specific protease family and is involved in the protein deubiquitination of histone or nonhistone proteins. USP22 was considered to be involved in many cancer types as an oncogene-like protein (23–29). In a variety of tumors, a high-expression of USP22 was thought to be associated with poor survival (30–32). Based on the findings of these studies, some researchers have attempted to evaluate the potential prognostic value of USP22 expression in GC patients. However, there is still no consensus on the OS of GC patients with USP22 expression. Lim et al. reported that no significant difference was found in the 5-year survival rate between USP22-negative and USP22-positive patients. However, some studies have shown that GC patients with an overexpression of USP22 have poorer survival rates than those with a low expression of USP22 (9, 10, 12, 13). In our study, the summarized results suggested that patients with an overexpression of USP22 tend to have a poor OS. The results were further validated in the TCGA database and then survival analysis was performed using the Kaplan–Meier plotter.

Valuable evidence in our meta-analysis has been provided for determining the relationship between USP22 expression and clinicopathologic parameters and prognosis of GC patients, which is helpful for clinical decision-making and promoting related research. Nevertheless, several potential limitations existing in the current meta-analysis should be removed. First, although we searched many websites such as PubMed and web of science, the account of the included literature is still small, which may cause bias in our conclusions. Second, the research groups are relatively small and are mostly from China, which may lead to a reduction of universality and heterogeneity. Furthermore, due to the unavailability of individual information, we could not consider some confounding factors such as smoking, surgery type and other environmental factors. In order to eliminate these limitations, high-quality studies are urgently needed in future work.

## Conclusion

In conclusion, this meta-analysis demonstrates that USP22 expression is higher in GC tissues than in normal tissues. USP22 expression is associated with lymph node metastasis, distant metastasis, TNM stage and poor OS of GC patients but is not associated with age, gender, depth of invasion, tumor differentiation and tumor size. Database validation and bioinformatics analysis verify that USP22 may be an indicator of poor prognosis in GC patients. The findings of this study suggest that USP22 may be a potential poor prognostic marker of GC patients.

## Data Availability

The original contributions presented in the study are included in the article/**Supplementary Material**, and further inquiries can be directed to the corresponding author/s.
