# Supplementary material for: Clinicopathological and Prognostic Value of USP22 Expression in Gastric Cancer: A Systematic Review and Meta-Analysis and Database Validation
Source: Front Surg. 2022 Jun 16;9:920595. doi: 10.3389/fsurg.2022.920595 (PMC9243499; doi:10.3389/fsurg.2022.920595)
Supplement: Supplementary file 1 [file Table_3_v1.doc]

**Supplementary Figure 1**





Supplementary Figure 1. Subgroup analysis between USP22 expression and overall survival of GC patients. (a) the relationship between USP22 and different N stage of GC patients. (b) the relationship between different TNM stage of GC patients. (c) the relationship between different M stage of GC patients.
